# Dynamically tunable second-harmonic generation using hybrid nanostructures incorporating phase-change chalcogenides

**DOI:** 10.1515/nanoph-2022-0051

**Published:** 2022-04-26

**Authors:** Muliang Zhu, Sajjad Abdollahramezani, Chentao Li, Tianren Fan, Hayk Harutyunyan, Ali Adibi

**Affiliations:** School of Electrical and Computer Engineering, Georgia Institute of Technology, 778 Atlantic Drive NW, Atlanta, GA 30332, USA; Department of Physics, Emory University, 400 Dowman Drive, Atlanta, GA 30322, USA

**Keywords:** gap-surface plasmon, phase-change chalcogenides, second-harmonic generation

## Abstract

Nonlinear metasurfaces with high conversion efficiencies have been vastly investigated. However, strong dynamic tunability of such devices is limited in conventional passive plasmonic and dielectric material platforms. Germanium antimony telluride (GST) is a promising phase-change chalcogenide for the reconfiguration of metamaterials due to strong nonvolatile changes of the real and imaginary parts of the refraction index through amorphous-crystalline phase change. The orderly structured GST has an even higher potential in tunable second-harmonic generation (SHG) with a non-centrosymmetric crystal structure at the crystalline phase, while the amorphous phase of GST does not exhibit bulk second-order nonlinearity. Here, we experimentally demonstrate SHG switches by actively controlling the crystalline phase of GST for a GST-based hybrid metasurface featuring a gap-surface plasmon resonance, and a quarter-wave asymmetric Fabry–Perot (F–P) cavity incorporating GST. We obtain SHG switches with modulation depths as high as ∼ 20 dB for the wavelengths at the on-state resonance. We also demonstrate the feasibility of multi-level SHG modulation by leveraging three controlled GST phases, i.e., amorphous, semi-crystalline, and crystalline, for the gap-surface plasmon hybrid device, which features stronger light–matter interaction and has higher resonant SHG efficiencies than the asymmetric F–P cavity device at respective GST phases. This research reveals that GST-based dynamic SHG switches can be potentially employed in practical applications, such as microscopy, optical communication, and photonic computing in the nonlinear regime.

## 1 Introduction

Second-harmonic generation (SHG) [1, 2] is widely employed in conversion of an optical signal to shorter wavelengths using non-centrosymmetric media [3] and second-order surface nonlinearity [4], originated from the symmetry breaking of the inherent crystal structure of a material [3] and large refractive index differences of materials on the two sides of an interface [5], respectively. This poses special requirements for materials or material interface selections to realize efficient SHG. Nevertheless, SHG is more frequently used than third-harmonic generation (THG) for generating light with shorter wavelengths, because SHG is a stronger effect, and thus, more efficient than THG [6]. Ultracompact SHG sources demand nanophotonic structures to manipulate light at subwavelength scales with extraordinary optical properties [7] beyond those achieved in bulk materials in addition to strong second-order nonlinearities. This can even extend the wavelength range of converted light to deep ultraviolet [8]. Tunable nanophotonic SHGs are typically realized by (1) all-optical modulation induced by Kerr-type coupled resonance shifting [9] and transient symmetry breaking [10] of passive nanostructures, or (2) using an external stimulus to tune the second harmonic (SH) signal from the nanostructures in real time [11, 12]. However, the reconfigurability of these structures is limited by the relatively small tunability of the optical nonlinearity in passively or actively tunable material configurations.

With fast and large refractive index tunability, phase-change materials (PCMs) [13], [14], [15], [16] have recently been widely studied for optical reconfigurability in the linear regime and subwavelength scale. GST (germanium antimony telluride, Ge_2_Sb_2_Te_5_), as one of the most widely used PCMs, has garnered widespread interest in nanophotonics [17], [18], [19], [20]. The real and imaginary parts of the refractive index of GST can experience changes by over 50% and 10 times, respectively, when switching between amorphous (a-GST) and crystalline (c-GST) phases through precisely controlled heating. GST exhibits other extraordinary characteristics, such as down-to-nanometers scalability, reasonably fast (down to hundreds of nanoseconds) switching times, highly robust switching up to 10^12^ cycles, power-efficient nonvolatility, high thermal stability, CMOS-technology and on-chip-integration compatibility, etc. [17, 21]. GST has been extensively studied in functional linear plasmonic devices [22]. However, the relatively high optical loss of GST during crystallization, especially at visible and near-infrared (NIR) wavelengths, is a major obstacle for the performance of GST-based nonlinear optical devices. Alloys of GST like Ge_2_Sb_2_Se_4_Te_1_ (GSST) [23] can provide considerably lower optical losses at these wavelengths. Yue et al. demonstrated mid-infrared (MIR)-excited THG with a very high efficiency by patterning arrays of subwavelength GSST cylinders supporting a magnetic dipolar resonance [24]. However, using GSST has the shortcoming of a relatively slow response of the phase change mechanism.

In terms of nonlinear reconfiguration, THG modulation is mostly reported for metamaterials incorporating GST, where both a-GST and c-GST exhibit third-order nonlinearity. The refractive index, loss, and third-order nonlinear optical susceptibility (*χ* ^(3)^) of a-GST are substantially smaller than those of c-GST [25]. Experimental studies of THG amplitude [25] and band [26] modulations with asymmetric subwavelength Fabry–Perot (F–P) cavities have been showcased by incorporating GST as the dominant *χ* ^(3)^ medium. Patterned nanostructures incorporating GST for THG modulations mostly appear in reported theoretical studies due to challenges with associated fabrications. A gap-surface plasmon resonance structure exhibiting an enhanced THG nearfield intensity modulation incorporating GST as the *χ* ^(3)^ medium [27], and a giant switching design of THG from an all-dielectric Fano-resonant metasurface modulated by an asymmetric F-P cavity incorporating GST [28], have been reported. More recently, the increased loss at the c-GST phase is utilized [29] to exhibit THG off-state hybridizing with a Fano-resonant amorphous silicon metasurface, while the THG response at the less lossy a-GST phase represents the on-state. In contrast, fewer SHG modulation demonstrations have been reported with GST. The potential for SHG modulation with GST phase change was indicated by a study of tunable grain orientation of GST thin films by exhibiting substantially contrasted SHG responses before and after annealing [30]. A theoretical study leverages GST phase change for manipulating SHG response by adjusting the nearfield in lithium niobate (LiNbO_3_) nano-resonators in a GST/LiNbO_3_ hybrid device [31]. The manipulation principle is based on distinct losses of GST phases of different crystallinity as revealed in Ref. [29].

In this paper, we seek to leverage metal-GST-metal gap-surface-plasmon resonance (compared with an asymmetric F-P GST nanoscale cavity) and multiple crystallinity phases of GST (a-GST, semi-c-GST, and c-GST) to demonstrate efficient three-level SHG switching using the potential high contrast of second-order nonlinear optical susceptibility *χ* ^(2)^ between a-GST and c-GST phases. The design and fabrication of the target structures are covered in Section 2. Results are presented and discussed in Section 3. Final conclusions are made in Section 4. The settings of experimental characterization are described in Section 5.

## 2 Design and fabrication

**Figure 1:**
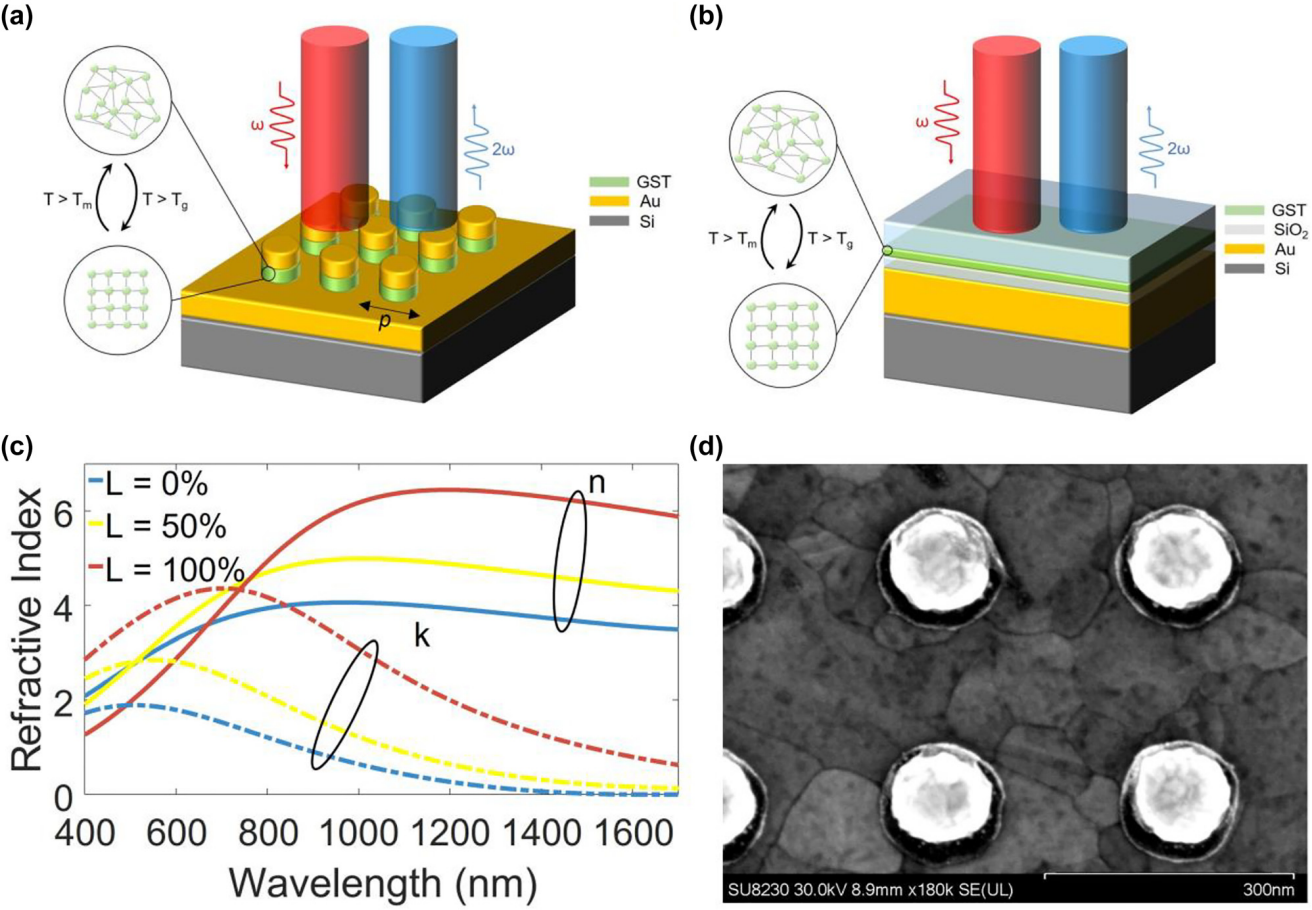
Device structures and refractive indices of the functional material. (a) Schematic of the Au-GST-Au device composed of an array of Au (35 nm-thick)/GST (25-nm-thick) nano-disks on an Au reflector (100 nm-thick) and a silicon substrate. The diameter of the nano-disk is selected as 120 nm, and the period of the metasurface is marked as *p*. (b) Schematic of the asymmetric F–P structure to be compared with the Au-GST-Au device. The Au reflector is also 100-nm thick, the bottom SiO_2_ is 25 nm thick, the GST is 20 nm thick, and the top SiO_2_ is 50 nm thick. *T*_g_ and *T*_m_ are the glass and melting temperatures of GST. (c) Real (solid curves) and imaginary (color-correspondent dashed curves) parts of the refractive index of the 3 different crystalline phases of GST. *L* represents percentage crystalline content of GST. (d) A top-view SEM image of several unit cells of a fabricated Au-GST-Au structure with a scaling measure marked.

The hybrid GST device featuring gap-surface-plasmon resonance is composed of an array of gold (Au)/GST nano-disks on an Au reflector, as shown in Figure 1(a). The Au reflector is set to be 100 nm thick to prevent light transmission at the NIR and visible wavelengths. The Au/GST nano-disk comprises a 25 nm-thick GST layer and a 35 nm-thick Au layer. The diameter of the nano-disk is selected as 120 nm, and the considered periods *p* of the metasurface are 220 nm, 250 nm, and 280 nm. The schematic of the asymmetric F–P device is illustrated in Figure 1(b). To avoid fabrication complications, our device is designed with four layers. A layer of Au as a back reflector is first deposited on a Si substrate. The Au layer is followed by a sandwiched structure of silicon dioxide (SiO_2_)-GST-SiO_2_. The thickness of the GST layer is set to 20 nm. The thickness of the bottom SiO_2_ layer is set to 25 nm, which is optically thick enough to guarantee the coherent interference of the incident and reflected linear signals at the GST layer. In addition, the bottom oxide/GST layers form a quarter-wave cavity on Au for a fundamental wavelength (*λ*_f_) of interest ∼ 1150 nm, which can exhibit substantial field interference in the dielectric layers featuring absorption resonance [32], despite the high loss of GST at *λ*_f_ ∼ 1150 nm. The top 50 nm-thick SiO_2_ layer serves as a capping layer to protect the GST layer from oxidation and provides a transmission window for SHG emission. We conduct linear simulations using the finite element method (FEM) through the full-wave electromagnetic modeling software COMSOL Multiphysics. Since the GST surface is oxidized upon immediate exposure to air [33], a GST oxide layer of 10 nm is considered at the top interface (i.e., between GST and Au) of the Au-GST-Au nanostructure as well as on the sidewalls. No GST oxide is added to the bottom interface for this nanostructure (i.e., between the bottom Au reflector and GST) since the deposition of GST on Au does not expose the GST at that interface with air. We also considered a 5 nm-thick GST oxide at the GST/top oxide interface for the asymmetric F–P cavity structure. These GST oxide thicknesses are selected based on previous experience in comparing theory and experiments while working with GST-based metasurfaces. We believe that with the smaller surface-to-volume ratio of the F–P structure, the oxidation effect is reduced compared to that in the Au-GST-Au nanostructure. Refractive indices of the amorphous and fully crystalline phases were obtained from ellipsometry of sputtered GST films on prime silicon substrates at the Georgia Tech cleanroom. We resorted to Lorentz-Lorenz formula [34] to describe the complex refractive index of GST in the semi-crystalline phase. The real and imaginary parts of refractive indices of a-GST (*L* = 0%), semi-c-GST (*L* = 50%), and c-GST (*L* = 100%) are plotted in Figure 1(c), where *L* represents the crystallinity percentage of GST. For the sake of simplicity, the GST oxide layers are modeled as a non-dispersive lossless material with an assumed refractive index of 2.5. The refractive index of Au is adopted from Johnson and Christy’s experimental data [35]. For the sake of simplicity, we only study plane-wave excitations to the structures with normal incidence from the air as shown in Figure 1(a) and (b).

We fabricated the as-grown Au-GST-Au structures by electron-beam (e-beam) evaporation of the Au reflector, patterning of e-beam lithography resist, RF magnetron sputtering of GST, the e-beam evaporation of the top Au layer on a prime silicon substrate, and lift-off. The samples were encapsulated by ∼ a 10 nm-thick layer of SiO_2_ deposited using atomic layer deposition (ALD) at ∼100 °C (a safe temperature for SiO_2_ deposition while preventing GST crystallization, which begins at *T*_g_ ∼ 135 °C [13]) immediately after the lift-off process to protect GST from oxidation. Figure 1(d) shows a top-view scanning electron microscopy (SEM) image of several unit cells of an Au-GST-Au structure with representative dimensions marked. The GST nano-disks exhibit radii slightly larger than the Au nano-disks due to the slight shadowing effect by the patterned e-beam resist during e-beam evaporation of the top Au layer. The non-a-GST samples were obtained with controllable partial and full annealing to semi-c-GST and c-GST phases on a hotplate, respectively. We fabricated the as-grown asymmetric F–P cavity samples by consequent depositions of the Au reflector, bottom SiO_2_, GST, and top SiO_2_ on a prime silicon substrate. The SiO_2_ layers are e-beam evaporated. The e-beam evaporation processes were conducted with a Denton Explorer system. The GST sputtering processes were conducted with the Chalcogenide Materials Sputterer. The e-beam lithography process was conducted with an Elionix ELS-G100 system. The ALD process was conducted with the Cambridge NanoTech Plasma ALD. Only the extreme crystalline phases are considered for the asymmetric F–P cavity samples as a control study group for the Au-GST-Au samples. The c-GST F–P cavity sample was obtained with full annealing to the c-GST phase on the hotplate at the same condition as that led to the crystalline Au-GST-Au samples.

## 3 Results and discussion

**Figure 2:**
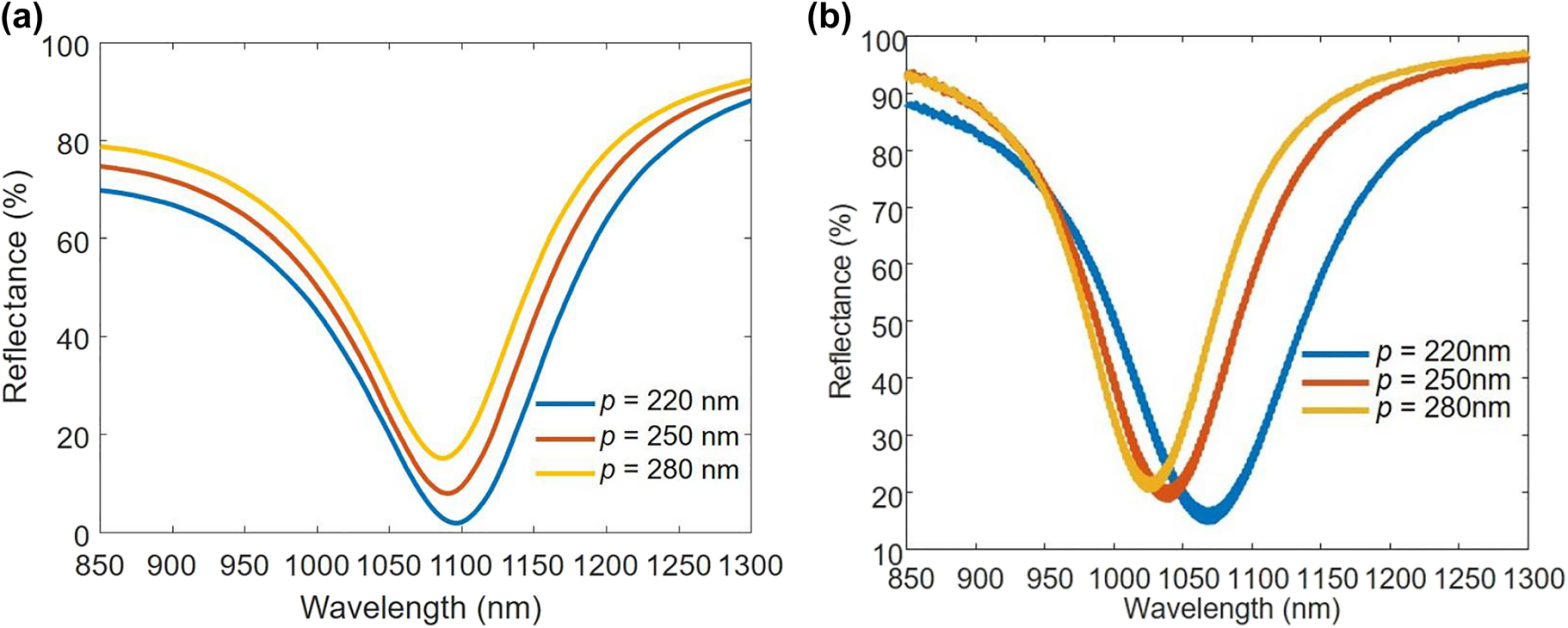
Linear responses at different metasurface periods. (a) Simulated and (b) measured reflectance spectra for the structure in Figure 1(a) with cases of *p* = 220 nm, *p* = 250 nm, and *p* = 280 nm at the a-GST phase.

Simulated and measured linear reflectance spectra of the structure in Figure 1(a) with periods *p* = 220 nm, 250 nm, and 280 nm for the a-GST phase are shown in Figure 2(a) and (b). There is a general blue-shift of the measured resonances at different values of *p* compared to the simulation results, and the measured resonances cover a wider wavelength range compared to the simulated resonances. This is possibly due to fabrication imperfections including the error caused by shadowing effect indicated in Figure 1(d). The resonance blue-shifts with increasing *p* since the effective index of the structure (considering equivalent uniform layers) decreases, with no change in the Au/GST nano-disk dimensions. The absorption of the structure at resonance decreases with increasing *p* since the ratio of lossy Au/GST nano-disk area to the total metasurface area decreases. The structure at non-a-GST phases is expected to exhibit a red-shifted resonance compared to the a-GST phase since the refractive index of GST increases with crystallinity. Considering the range of resonances for different values of p, we selected Au-GST-Au metasurfaces with *p* = 220 nm for SHG in the rest of our designs and experimental investigations. This is primarily due to the limitation in the characterization system, which uses an optical parametric oscillator (OPO) with limited stability at excitation wavelengths <1050 nm.

**Figure 3:**
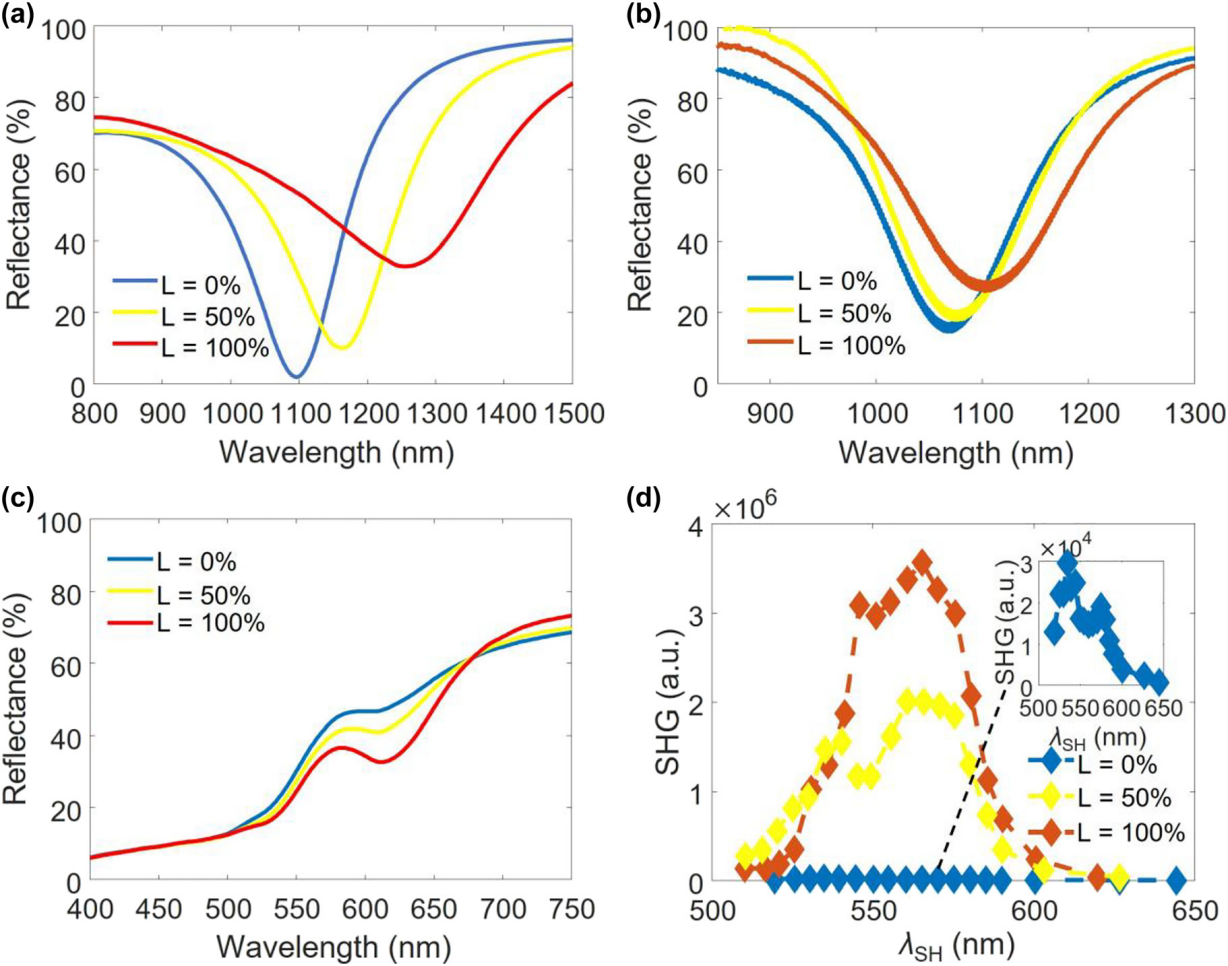
Spectral responses of the hybrid metasurface at GST phases of different crystallinity. (a) Simulated and (b) measured reflectance spectra for the structure in Figure 1(a) with *p* = 220 nm at investigated GST phases. (c) Simulated reflectance spectra of the structure with parameters for Figure 3(a) at the visible wavelengths. (d) Measured SHG response of the correspondent fabricated samples, where *λ*_SH_ represents the SHG wavelength, and the inset shows a zoomed-in view of the weak SHG signal for the a-GST case.

Simulated and measured linear reflectance spectra of the structure designed at the NIR *λ*_f_ *s* in Figure 1(a) with period *p* = 220 nm for all investigated GST phases are shown in Figure 3(a) and (b), respectively. There is a general blue-shift of the resonances at the investigated GST phases compared to those of the simulations, and the measured resonances are closer to each other compared to the simulated resonances possibly due to fabrication imperfections. The gradually broadened reflection ‘dip’ is observed with increasing *L* due to the higher GST loss at higher values of *L*. Figure 3(c) shows the simulated linear reflectance spectra of the structure with parameters for Figure 1(a) at the visible wavelengths. There is a resonance ‘dip’ with a depth that starts from ∼0 at the a-GST phase and significantly increases (i.e., stronger resonance) with GST crystallinity. In addition, the resonance exhibits a much smaller redshift with increased GST crystallinity, compared to the designed resonance at the NIR. And the resonance wavelength is slightly longer than the SH at the NIR resonance, considering both simulated results in Figure 3(a) and measured results in Figure 3(b). Figure 3(d) presents measured reflected SHG intensity spectra of the device for the investigated GST phases. One arbitrary unit (a.u.) in Figure 3(d) is estimated to correspond to an SHG efficiency of ∼7 × 10^−12^ at a pump power density of ∼0.1 GW/cm^2^. This a.u. applies to all SHG efficiencies in this article. As shown in the inset of Figure 3(d), the SHG peak intensity for the device at *L* = 0% (i.e., a-GST) is observed at the SHG emission wavelength of ∼535 nm, which approximately matches the linear reflection ‘dip’ of *λ*_f_ (∼1070 nm from Figure 3(b)). There is an SHG peak of smaller intensity at ∼575 nm possibly due to the resonant mode at the SH wavelength indicated in Figure 3(c) that can overcome the reduced signal at the *λ*_f_. The SHG peak intensities of the device for *L* = 50% and *L* = 100% are ∼50 and ∼100 times higher than that for *L* = 0%, respectively, although the quality factor (*Q*) of linear resonance is reduced with GST crystallization. This clearly indicates that the second-order nonlinearity of c-GST is much stronger than the surface second-order nonlinearity of Au in this device. Note that the SHG peaks at ∼575 nm for the non-amorphous GST phases (see the yellow and red curves in Figure 3(d)) are higher than the peaks corresponding to the linear resonances, which indicates that the hybridization of the linear mode with the SH resonant mode becomes stronger than the peak of non-hybridized linear mode with GST crystallization. The observation is in accordance with the strengthened resonance around SH wavelengths, which is indicated in Figure 3(c). This device, to the best of our knowledge, exhibits the largest extinction ratio of SHG switching for a nonlinear metasurface reported to date. In addition, by using different values of *L* (different levels of GST crystallinity), we can modify both the amplitude and the wavelength of the maximum SH signal. We attribute this to the strong tunability of the GST nonlinearity with its change of crystallinity (*L*). This makes the platform in this paper an unmatched solution for other applications of metasurfaces for second-order optical nonlinearity.

**Figure 4:**
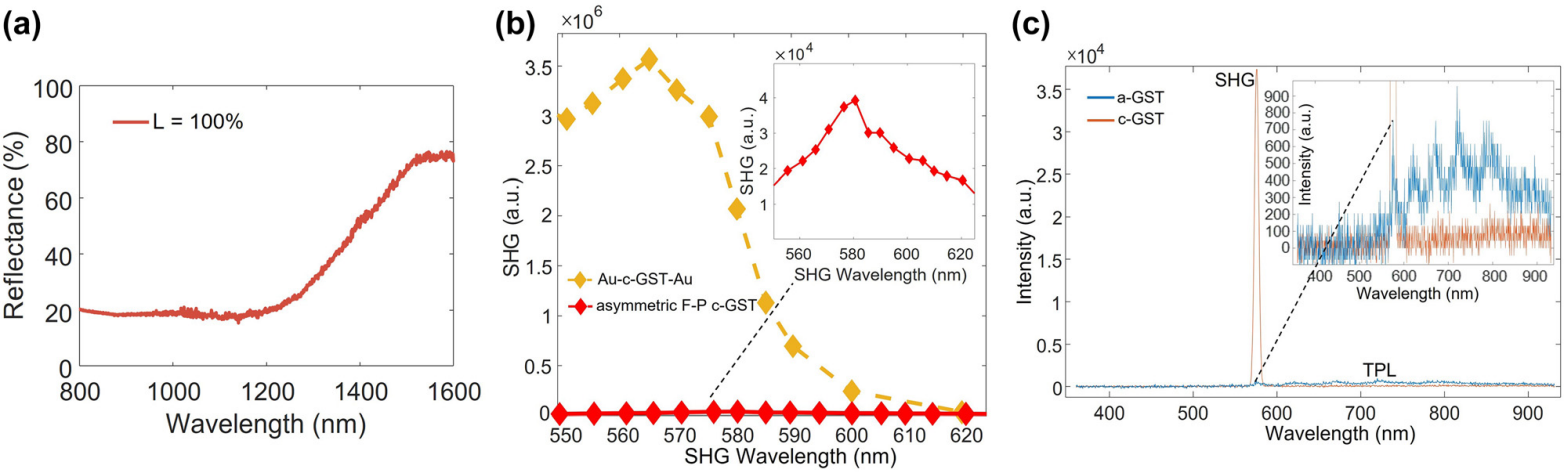
Measured (a) linear reflectance spectrum for the structure in Figure 1(b) at the c-GST phase (L = 100%), (b) c-GST phase SHG spectra of the structure in Figure 1(a) with *p* = 220 nm, and the structure in Figure 1(b), (c) reflected nonlinear emission intensity spectra, at the visible wavelengths, of the asymmetric F–P structure for the a-GST and c-GST phases at a pump wavelength of 1150 nm. TPL: Two-photon luminescence.

Using the observations for the SHG in the structure in Figure 1(a), we designed the asymmetric F–P GST device in Figure 1(b) by selecting its geometric dimensions to experimentally match the SHG resonance wavelength from the F–P device to that from the Au-GST-Au patterned structure at the c-GST phase (to achieve a substantial second-order nonlinearity). The measured linear reflectance spectrum of the fabricated F–P structure for the c-GST phase is shown in Figure 4(a). The reflectance band is much broader than that of the Au-c-GST-Au patterned structure. This is due to the fact that the absorption resonance of a loss mechanism [32] is much weaker than the gap-surface-plasmon resonance with high field confinement. Figure 4(b) shows a comparison plot of measured SHG spectra at the c-GST phase of the selected structure in Figure 1(a) with *p* = 220 nm and the structure in Figure 1(b). As shown in the inset of Figure 4(b), the SHG peak intensity for the F–P device with the c-GST phase is observed at the SH emission wavelength of ∼575 nm, which approximately matches the shallow reflection ‘dip’ (∼1150 nm) at the *λ*_f_ in Figure 4(a). The SHG resonance wavelength of the Au-c-GST-Au patterned structure is approximately the same as that of the F–P c-GST structure. However, it is clear from Figure 4(b) that the SHG output intensity of the Au-c-GST-Au patterned structure is ∼100 times that of the F–P c-GST structure given that their GST thicknesses are similar. This is additional evidence for the strength of the gap-surface-plasmon resonance, which can mitigate the relatively high loss of GST, especially for its most lossy (i.e., crystalline) phase. Figure 4c shows measured reflected nonlinear SHG emission intensity spectra, at the visible wavelengths, of the asymmetric F–P structure in Figure 1(b) for the a-GST and c-GST phases at the pump wavelength of 1150 nm. The output SHG peak intensity of the F–P structure for the c-GST phase is measured to be ∼50 that of the a-GST case, showing the possibility of ON/OFF switching of the SH signal. This modulation depth is lower than that of the Au-GST-Au structure (∼100), which might be due to greater susceptibility of the relatively weak SHG signal to the background noise for the F–P structure. As shown in the inset of Figure 4(c), a heavy tail of the SHG emission spectrum at the a-GST phase (extending to longer spectral wavelengths than the SHG emission) represents the two-photon luminescence (TPL) following the two-photon absorption process, which is a nonlinear optical process related to the imaginary part of the *χ* ^(3)^s of nonlinear materials [36]. It is evident that the TPL at the c-GST phase of the F–P structure is generally weaker than that observed at the a-GST phase, though the imaginary part of *χ* ^(3)^_c-GST_ is generally larger than that of *χ* ^(3)^_a-GST_ at the NIR wavelengths according to the empirical Miller’s rule, i.e., *χ* ^(3)^ ∝ (*χ* ^(1)^)^4^, where *χ* ^(1)^ is the first-order optical susceptibility of the material [37]. The weaker TPL of the c-GST structure can be due to a smaller linear nearfield and larger absorption of the intrinsic TPL signal in the more lossy c-GST phase.

**Figure 5:**
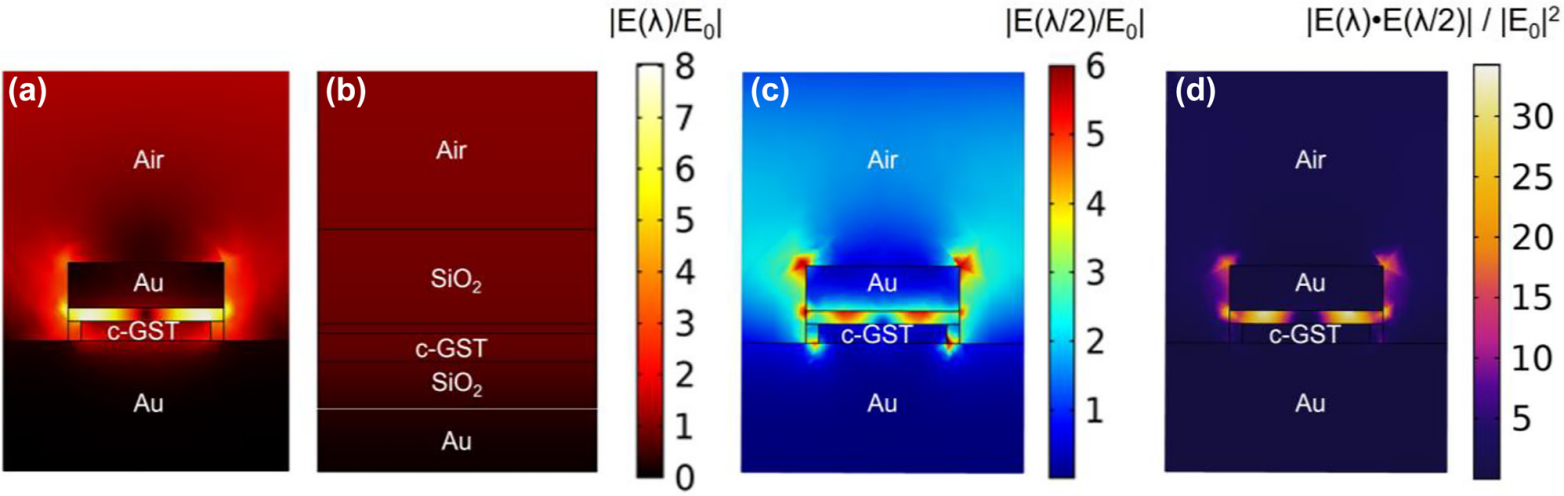
Middle cross-sectional plots of simulated normalized nearfields in a plane parallel to the polarization direction (i.e., the incident polarization is in the place of this figure) in (a) Figure 1(a) and (b) Figure 1(b) for the c-GST phase at *λ* = 1260 nm. The fields are normalized to the incident field. Figures 5(a) and (b) are in the same scale, and the maximum value of the scale bar is set to the maximum normalized nearfield in the Au-c-GST-Au structure at *λ* = 1260 nm. Figure 5(c) shows the normalized nearfield plot for the incidence wavelength of *λ*/2 (i.e., SH wavelength) under the same way of excitation. The mode overlap between nearfields in Figure 5(a) and (c) is plotted in Figure 5(d) in terms of normalized inner-product of the nearfields to the square of incident field. Solid lines show the extent of all materials. All material components of the structures are marked in the plot except for the GST oxide, which is represented by unmarked areas (on top of the c-GST layer) in the plots.

To better illustrate the sharp difference between SHG from the Au-c-GST-Au gap-surface-plasmon resonance and asymmetric F–P c-GST absorption resonance, we show in Figure 5(a) and (b) the middle cross-sectional plots of simulated normalized nearfields in a plane parallel to the polarization direction in Figure 1(a) and (b), respectively, at the c-GST phase at *λ*_f_ = 1260 nm. This corresponds to the simulated gap-surface-plasmon resonance of the structure in Figure 1(a) and approximate simulated absorption resonance of the structure in Figure 1(b). From Figure 5, we can observe that although the largest nearfield enhancement in the Au-c-GST-Au structure lies in the GST oxide between the lossy GST and the top Au layer (i.e., top GST-oxide layer, see Figure 5(a)), the nearfield enhancement in c-GST in Figure 5(a) is still substantially larger than that in Figure 5(b). Note that due to the large resonance nearfield on the Au/dielectric interfaces (as clearly seen from Figure 5(a)), the surface *χ* ^(2)^ of Au cannot be ignored as a component in the SHG sources of the Au-c-GST-Au structure. Thus, both surface *χ* ^(2)^ of Au and bulk *χ* ^(2)^ of c-GST coupling to the strong linear field interference contribute significantly to the largely enhanced SHG from the patterned Au-c-GST-Au structure compared to the asymmetric F–P c-GST cavity structure. In addition, we studied the simulated nearfield at the corresponding SH wavelength (i.e., 630 nm) and the mode overlap of the nearfields at the fundamental and SH wavelengths. Figure 5(c) shows the normalized nearfield plot at the SH wavelength. The mode overlap between nearfields in Figure 5(a) and (c) is plotted in Figure 5(d) in terms of normalized inner-product of the nearfields to the square of the incident field at the pump wavelength. It is clear from Figure 5(c) that the largest nearfield enhancement in the Au-c-GST-Au structure also lies within the top GST oxide layer, except for some hotspots on interfacial corners of the Au/c-GST nano-disk. Figure 5d shows a large mode overlap in the top GST oxide layer as well. Therefore, surface *χ* ^(2)^ of c-GST, which can couple efficiently with the mode overlap, might also be much larger than that of a-GST. The relative contributions of bulk *χ* ^(2)^ of c-GST, and surface *χ* ^(2)^s of Au and GST at different crystallinities to the SHG outputs in this structure worth further studying.

## 4 Conclusions

We experimentally demonstrated a new platform based on a metal-insulator-metal (MIM) device for efficient tunable SHG by integrating conveniently reconfigurable phase-change material GST with a plasmonic nanostructure. We compared the performance of the structure to a counterpart asymmetric F–P GST cavity structure, which has been studied before for nonlinear optical effects. Both structures are in subwavelength scale and exhibit SHG switching with ON/OFF ratios of ∼10^2^. The Au-GST-Au structure exhibits a much higher average SHG efficiency than the F–P GST structure among investigated GST phases. This is caused since the MIM structure features a gap-surface plasmon resonance with a stronger photonic interaction than the absorption resonance in the F–P structure. This study expands the linear optical intensity switching nanodevices to the SH paradigm and paves the way for more sophisticated designs for improved efficiency and functionality, which might be applicable to SHG microscopy, communication, and computing. These structures can be combined with ultrafast and compact heating processes using indium tin oxide (ITO) as demonstrated in Ref. [38, 39], or robust metals (e.g., tungsten (W), demonstrated in Ref. [21]) as Joule micro-heaters to expand the platform with new features for electrically tunable chip-scale nonlinear optical devices. The structure here can also be a reliable platform for SHG switching with higher conversion efficiencies (1) at the longer infrared wavelengths where the optical loss of GST is minimal or (2) by using alloys of GST, e.g., Ge_2_Sb_2_Se_4_Te_1_ (in short, GSST) [23, 24], which provide less optical loss at the NIR wavelengths.

## 5 Experimental section

The linear characterizations were conducted with an incandescent white light source and an InGaAs linear-array detector coupled with a spectrometer. Reflectance spectra were normalized with the reflected power of control areas of 100 nm-thick Au on the substrates for the samples. The SHG characterizations were conducted with an OPO (∼1000–1600 nm tuning range) pumped by a Ti:Sapphire laser (150 fs pulse duration and 80 MHz repetition rate, Coherent Chameleon) and a Si charge‐coupled device. A 100× objective lens (numerical aperture = 0.95) was used for effective collection of SHG signals. These characterization settings were applied to all the samples of both Au-GST-Au nanostructures and asymmetric F–P GST structures.
